# Control of nanostructure and pinning properties in solution deposited YBa_2_Cu_3_O_7−x_ nanocomposites with preformed perovskite nanoparticles

**DOI:** 10.1038/s41598-019-42291-x

**Published:** 2019-04-09

**Authors:** Ziliang Li, Mariona Coll, Bernat Mundet, Natalia Chamorro, Ferran Vallès, Anna Palau, Jaume Gazquez, Susagna Ricart, Teresa Puig, Xavier Obradors

**Affiliations:** 1 Institut de Ciència de Materials de Barcelona, ICMAB-CSIC, Campus de la UAB, 08193 Bellaterra, Spain; 2 Departament de Química, Facultat de Ciències, Universitat Autònoma de Barcelona, 08193 Cerdanyola del Vallès, Catalonia Spain

## Abstract

Solution deposited YBa_2_Cu_3_O_7−x_ (YBCO) nanocomposites with preformed nanoparticles represent a promising cost-effective approach for superior critical current properties under applied magnetic fields. Nonetheless, the majority of YBCO nanocomposites with high nanoparticle loads (>20%) suffer from nanoparticle coalescence and degraded superconducting properties. Here, we study the influence of nanoparticle concentration (0–25% mol), size (5 nm–10 nm) and composition (BaHfO_3_, BaZrO_3_) on the generation of structural defects in the epitaxial YBCO matrix, key parameter for vortex pinning. We demonstrate that flash-heated superconducting nanocomposites with 20 mol% preformed BaHfO_3_ or BaZrO_3_ perovskite secondary phases feature discrete and small (7 nm) nanoparticles and high density of YBa_2_Cu_4_O_8_ (Y248) intergrowths. We identify a synergy between Y248 intergrowth density and small nanoparticles to increase artificial vortex pinning centers. Also, we validate the multideposition process to successfully increase film thickness of epitaxial nanocomposites with competitive critical currents I_c_ at 77 K.

## Introduction

The outstanding ability of YBa_2_Cu_3_O_7−x_ (YBCO) nanocomposite thin films to carry high critical currents at high magnetic fields offers an unprecedented opportunity to be used in rotating machinery and high field magnets applications^1–6^. However, a reliable manufacturing methodology for reducing the cost/performance ratio is required to speed up market penetration^6–9^. Chemical solution deposition (CSD) is a well-established low cost preparation route to obtain high quality epitaxial YBCO thin films and multilayers^10–15^. There is an essential difference in the growth mechanisms of CSD-based nanocomposite films as compared to most of the vacuum-based approaches, such as pulsed laser deposition (PLD) and metalorganic chemical vapor deposition (MOCVD)^16–20^. While in the latter there exists a simultaneous growth of the YBCO superconductor and the secondary phases, in CSD films both phases have a sequential nucleation and growth so the final microstructure of both types of nanocomposites deeply differ^15,21–23^. In the last ten years, a strong effort has been devoted to prepare CSD-based YBCO nanocomposite thin films generated by the spontaneous segregation of nanometric oxide secondary phases (ranging from simple binary oxides to perovskites and double perovskites) during the YBCO conversion process^21,22,24–28^. This approach relies on the use of complex metalorganic solutions including all the required  metalorganic salts to form the nanocomposite and during the growth process, the homogeneous nucleation of oxide nanoparticles advances the epitaxial growth of the YBCO matrix which englobes the nanoparticles at their growth front. One of the main goals has been to achieve an accurate control of the nanoparticle size, its homogeneous distribution within the YBCO matrix and the induced defect structure because all these parameters deeply influence the vortex pinning efficiency and the amount of percolating current. Of course the final goal was to generate nanoparticles with the mean diameter of the spontaneously segregated nanoparticles in the range of the coherent length (ξ) of YBCO but, up to now, this has been turned out very challenging^4,20,29,30^. Instead, the vortex pinning mechanism has been shown to be mostly dominated by the presence of highly strained areas in the YBCO matrix, i.e. nanostrain (ε)^24^. When the oxide nanoparticles are randomly oriented, they trigger the formation of nanoscale defects in the YBCO matrix, being the most relevant ones the YBa_2_Cu_4_O_8_ (Y248) intergrowths (extra Cu-O chain) which ultimately generate highly strained localized areas, nanostrain, where Cooper pair formation is suppressed^31–34^. Therefore, in this scenario, a tight control of nanoparticle properties (size, shape, load, reactivity and mismatch with YBCO) and Y248 intergrowth characteristics (density, extension and distribution) are of fundamental importance for optimizing vortex-pinning landscape. Although the CSD-based YBCO nanocomposites prepared from spontaneous segregation has been proved effective for enhanced vortex-pinning properties for a wide variety of compositions (BaZrO_3_, Ba_2_YTaO_6_, Y_2_O_3_, BaCeO_3_, BaSnO_3_)^26,35^, the control of nanoparticle size and distribution at high loads remains very challenging^22,36,37^. Recently, an ingenious strategy based on the spontaneous segregation approach and multiple coating/calcination steps has been reported to achieve high nanoparticle loads (>25 mol%) with tuned sizes in the small range of 7 nm, with remarkable superconducting performances^23^.

The development of a more versatile route to prepare CSD-based YBCO nanocomposites using already preformed nanoparticles (pn-nanocomposites) has opened many new opportunities to design the nanostructure and pinning properties of YBCO nanocomposites^38–42^. In this case, a colloidal solution of oxide nanoparticles with a well-defined size, shape and concentration is mixed with a precursor solution containing the Y-, Ba- and Cu- metalorganic salts. Research studies in the last few years identified that for this methodology, one of the most important property that the preformed nanoparticles have to possess is chemical stability during YBCO growth^41,42^. In this line, preformed perovskite nanoparticles with unreactive character, such as BaMO_3_ (M = Zr, Hf), are the most promising ones^43^.

The main focus of this work is to carry out a thorough study of the influence of large quantities of BaMO_3_ nanoparticles on the pn-nanocomposite structure and pinning performance. The use of a fast conversion process, based on a flash heating step, is here presented as a powerful strategy to achieve a synergetic effect between small nanoparticles and partial dislocations for enhanced pinning performances in applied magnetic fields.

## Methods

### Nanoparticle Synthesis

BaMO_3_ (M = Zr, Hf) nanoparticles with 5 nm and 10 nm of mean diameter were synthesized via solvothermal method. The reaction started from Ba(OH)_2_ and Zr(OBu)_4_ or Hf(OBu)_4_ as molecular precursors in C_6_H_14_O_4_(TEG)/C_2_H_6_O(EtOH) and NH_3_·H_2_O. The precursors were treated at 180 °C for 1–20 h in a sealed reactor. The concentration of well-dispersed BaZrO_3_ and BaHfO_3_ nanoparticles was adjusted by adding the required amount of solvent for a well-known total volume of colloidal solution reaching concentrations as high as 72 mmol^−1^ for BaZrO_3_ and 204 mmol^−1^ for BaHfO_3_^43–45^.

### Preparation of YBa_2_Cu_3_O_7−x_ nanocomposites

The YBCO-BMO (BMO, B = Ba, M = Zr and Hf) precursor solutions were prepared by introducing well-known aliquot of BMO colloidal solution to a TFA-YBCO^10^ precursor solution to prepare a wide range of BMO compositions, from 0 to 25 mol%. The YBCO-nanoparticles precursor solution was deposited on a 5–50 nm YBCO seed layer prepared on a 5 × 5 mm^2^ (100) LaAlO_3_ (LAO) single-crystal substrates. The seed layer consists of a pyrolyzed pristine YBCO layer of 25–50 nm thickness^46^. Subsequently, the pyrolyzed layers were crystallized at 750–820 °C for 150 min in N_2_ − 0.02% O_2_ with P_H2O_ = 23 mbar. Two different high temperature thermal treatments were investigated in this work. One, using a 25 °C/min heating ramp (conventional thermal annealing, CTA) and another one using a fast-processed 1200 °C/min heating ramp (flash heating process, FH)^47^. See Supporting Information Figure S1(a) for further details. Finally, the superconducting YBCO phase was obtained by oxygen annealing at 550 °C for 210 min. This process leads to a typical film thickness of 150 nm. To achieve thicker YBCO nanocomposite films (up to 350 nm), a multi-deposition process separated by intermediate pyrolysis steps was performed.

### Characterization methods

#### Preformed BMO nanoparticles

The morphological features and size were characterized by 200 kV JEOL 2011 High-Resolution Transmission Electron Microscopy (HRTEM) with a resolution point of 1.8 Å at 200 kV. The phase analysis was conducted from powder x-ray diffraction (XRD) pattern using a Rigaku diffractometer equipped with rotating anode and a Cu Kα source (λ = 0.154056 nm).

#### YBCO nanocomposites

The phase and texture analyses of the YBCO nanocomposites were performed from two dimensional 2D-XRD patterns acquired using a Bruker AXS General Area Detector Diffraction System (GADDS) diffractometer operating with Cu K_α_ radiation. Nanostrain (ε) was determined using Williamson-Hall (WH) method^48,49^ by analyzing the symmetric (00 l) 2θ Bragg diffraction integral breadth acquired in a Siemens D5000 diffractometer. In-plane and out-of-plane texture analysis were analyzed from the (103) YBCO phi-scan (ϕ-scan) and (005) YBCO rocking curve (ω-scan), respectively. The nanoparticle distribution, size and atomic scale defect landscape of YBCO nanocomposite films were studied by scanning transmission electron microscopy (STEM) using a FEI Titan 60–300 microscope equipped with an X-FEG gun, a CETCOR probe corrector and a Gatan TRIDIEM 866 ERS energy filter operated in STEM mode at 300 kV. The surface morphology of the YBCO-BMO nanocomposite films was investigated using a scanning electron microscopy (SEM, FEI Quanta 200 FEG). Critical current density (J_c_) and superconducting transition temperature (T_c_) were obtained inductively from hysteretic magnetization and low field zero field cooling (ZFC) magnetization measurements performed with a superconducting quantum interference device (SQUID) magnetometer (Quantum Design, San Diego, CA). The discrimination between weak and strong pinning centers was based on the J_c_(T) dependence measured at 7 T according to the weak collective and strong correlated defects models^21,34,50,51^.

## Results and Discussion

### Properties of the BaMO3 (M = Zr, Hf) colloidal solutions

**Figure 1:**
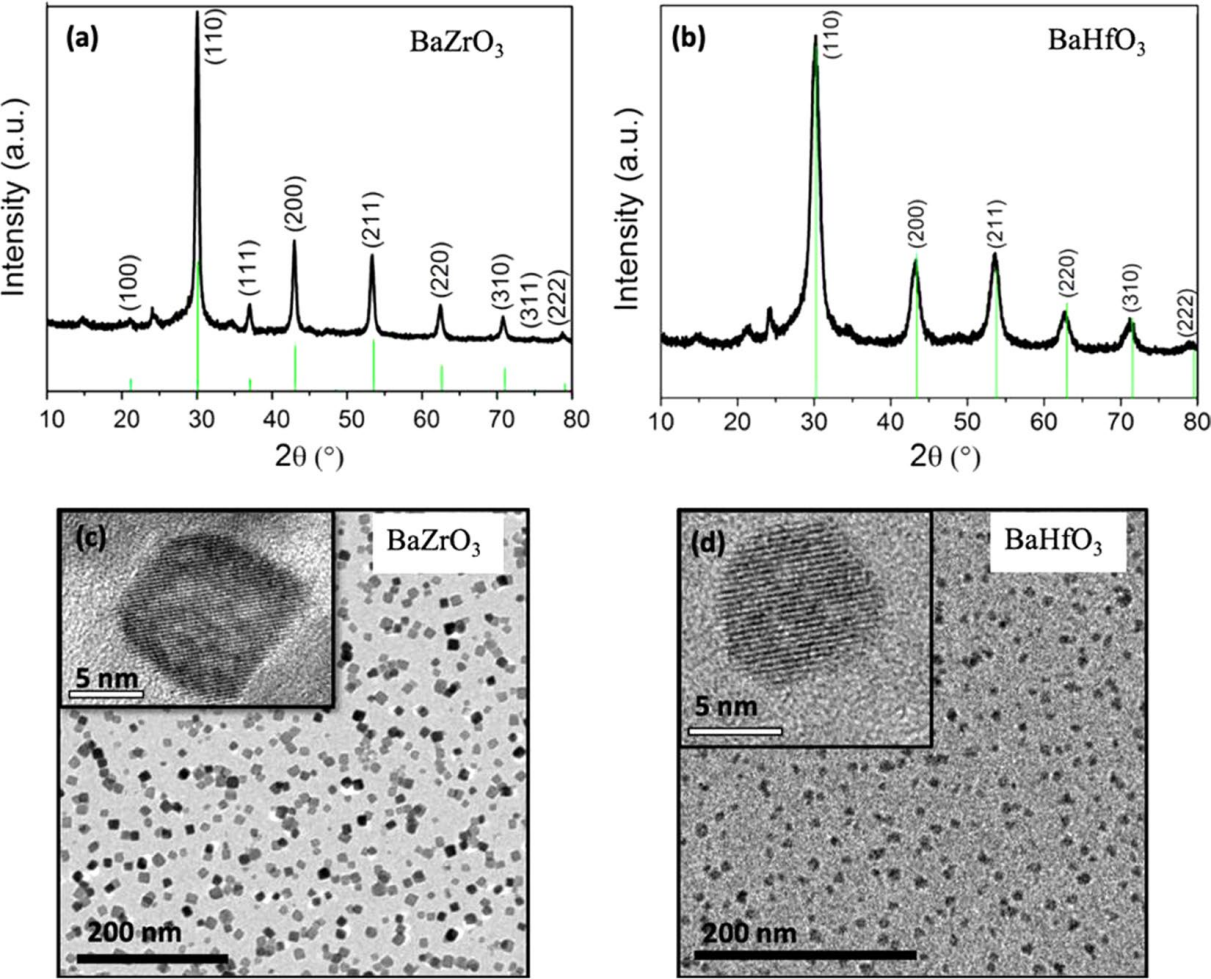
XRD powder patterns of solvothermal activated nanoparticles. (**a**) BZO, (**b**) BHO; TEM images of (**c**) BZO and (**d**) BHO nanoparticles. Inset: high resolution TEM image of the corresponding individual nanoparticle.

Phase and microstructural analysis of the as-synthesized nanoparticles is carried out by XRD and TEM, see Fig. 1. BaZrO_3_ (BZO) and BaHfO_3_ (BHO) preformed nanoparticles have both a cubic crystal structure with lattice parameters of a = 4.193 Å (JCPDS-ICDD 06-0399) and a = 4.171 Å (00-022-0084), respectively, see Fig. 1(a) and (b). TEM analyses reveal the formation of colloidal solutions of monodispersed nanoparticles without agglomerations. BZO preformed nanoparticles present a square-like shape whereas BHO nanoparticles are spherical (Fig. 1(c) and (d), respectively). Importantly, these alcoholic colloidal solutions have been successfully introduced into ionic TFA-YBCO precursor solution being stable for more than three weeks with nanoparticle loads as high as 25% mol. All these suspensions lead to homogeneous and crack-free pyrolyzed films, mandatory to fabricate high quality YBCO-BMO nanocomposite films (see Supporting Information Figure S1(b)).

### YBa_2_Cu_3_O_7−x_ Nanocomposites with preformed nanoparticles

#### Influence of BaZrO_3_ nanoparticle concentration on nanocomposite structure and physical properties

**Figure 2:**
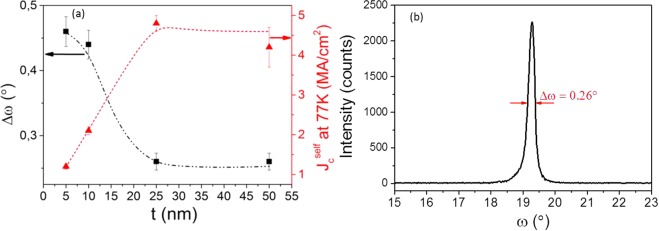
Analysis of the YBCO seed layer quality to grow YBCO-BZO pn-nanocomposites. (**a**) FWHM of XRD ω-scan YBCO (005) (black square) and ${{\rm{J}}}_{{\rm{c}}}^{{\rm{s}}{\rm{e}}{\rm{l}}{\rm{f}}}$ (77 K) (red triangle) dependence with the YBCO seed layer thickness (t) for YBCO-20 mol% BZO (10 nm) pn-nanocomposite films (**b**) Δω (005) YBCO of YBCO-20% BZO thin film.

As a first step, we study the influence of preformed BZO nanoparticle load with 10 nm initial mean diameter in the YBCO structure and physical properties, prepared following the conventional thermal process, CTA (Figure S1(a)). It has been recently demonstrated that the use of a pyrolyzed pristine YBCO seed layer prior to pn-nanocomposite deposition is a simple and ease approach to overcome the accumulation of preformed nanoparticles at the YBCO//LAO interface which subsequently perturb the biaxial texture of YBCO^41,43^. The critical nanoparticle load by which YBCO seed layer is mandatory to ensure c-axis YBCO oriented growth is 12 mol%. Below 12 mol%, the concentration of nanoparticles on the substrate surface is negligible and does not disrupt the heteroepitaxial growth (see Figure S2). To design a cost-effective process is important to identify the minimum seed layer thickness required for high quality nanocomposites. Full width at half maximum (FWHM) of (005) YBCO rocking curve (Δω) and self-field (${J}_{c}^{self}$) at 77 K were the chosen parameters to evaluate the YBCO pn-nanocomposite texture quality and superconducting performance, respectively. We aim to achieve the maximum J_c_ value for the minimum Δω. Thus, from Fig. 2(a) it is clearly seen that 25 nm is the threshold seed layer thickness to obtain nanocomposites with Δω (005) below 0.3° and ${J}_{c}^{self}$ at 77 K of ∼4.5 MA/cm^2^. Figure 2(b) shows the rocking curve from the (005) YBCO reflection for YBCO-20% BZO thin film. Therefore, from here on, seed layer thickness of 25–50 nm has been used.

**Figure 3:**
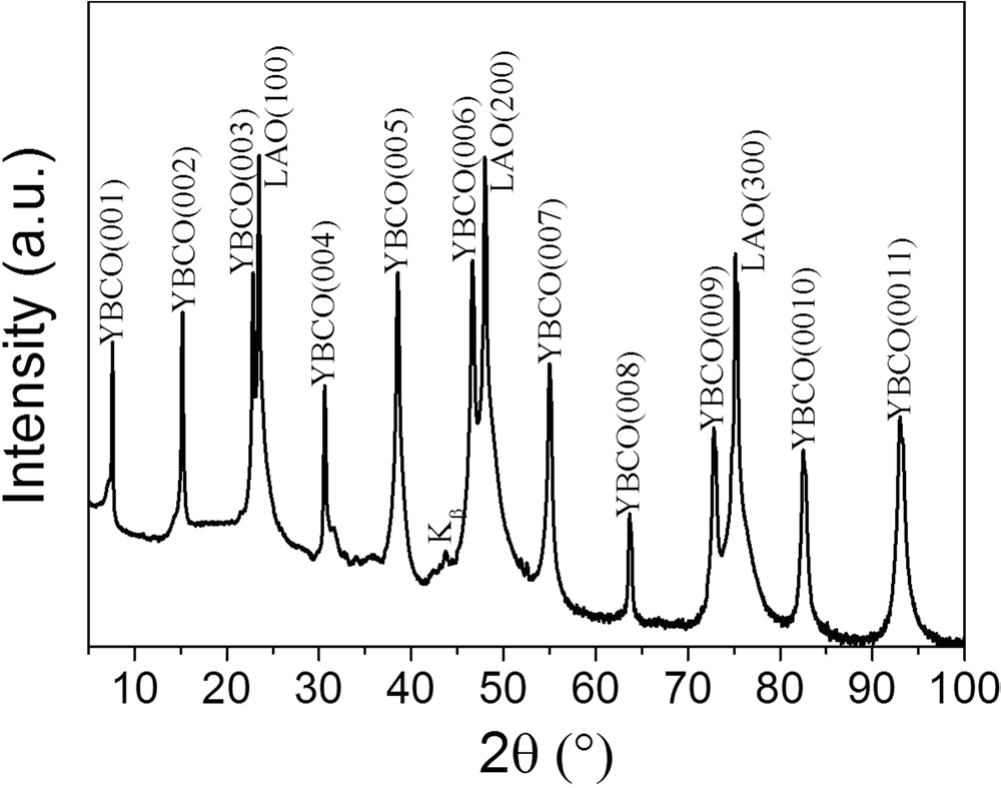
XRD θ-2θ scan of 150 nm YBCO-20% mol BZO pn-nanocomposite film grown on YBCO seed// LAO substrate by CTA.

YBCO precursor solutions with increasing BZO nanoparticle loads starting from 0 to 25 mol% were systematically deposited on YBCO seed//LAO. Figure 3 shows the XRD θ-2θ scan for 20 mol% BZO pn-nanocomposite where only (00 l) YBCO and (00 l) LAO Bragg reflections are identified, indicating c-axis oriented growth. Biaxial texture was further confirmed from in-plane (Δϕ(103)YBCO = 0.9°) and out-of-plane (Δω(005) YBCO = 0.3°) texture analysis (Figure S3). Importantly, epitaxial pn-nanocomposite films with BZO concentrations as high as 25 mol% can be obtained although for this nanoparticle load, slight texture degradation is devised, Δω(005) YBCO = 0.7°, (Figure S4).

**Figure 4:**
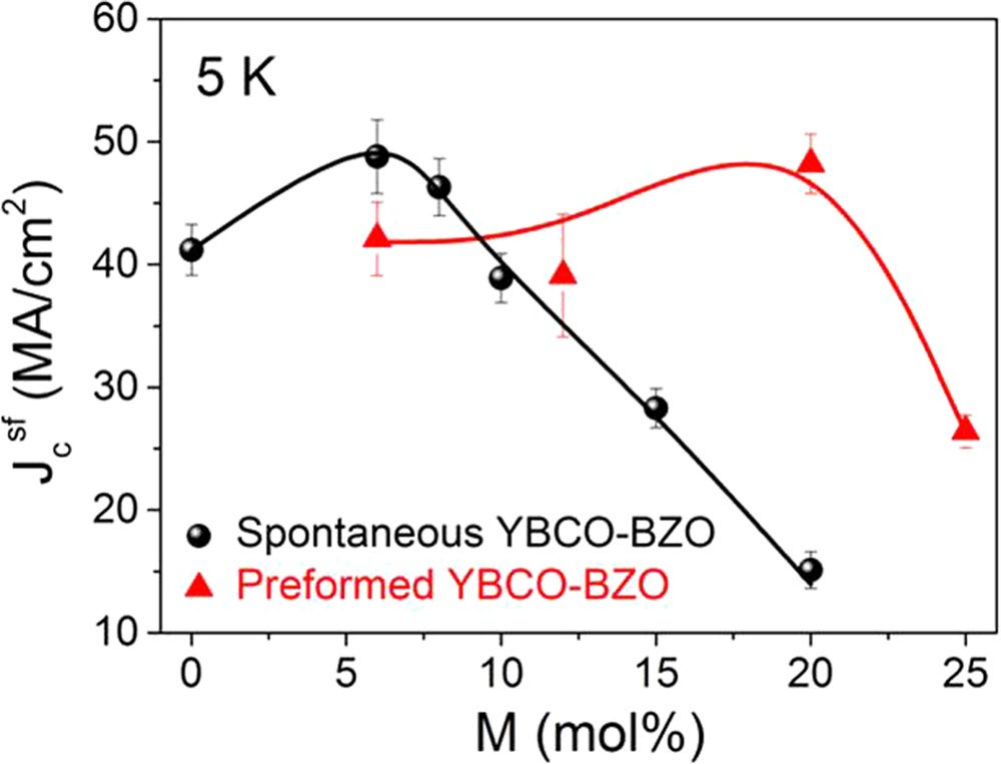
${{\rm{J}}}_{{\rm{c}}}^{{\rm{s}}{\rm{e}}{\rm{l}}{\rm{f}}}$ (5 K) dependence with nanoparticle molar concentration (M) of YBCO-BZO (10 nm) pn-nanocomposite films (red triangle) compared with spontaneously segregated YBCO nanocomposite films (black sphere). Error bars are determined from the fit uncertainty in the corresponding data analysis.

Figure 4 compares the ${J}_{c}^{self}$ (5 K) dependence on the BZO nanoparticle concentration in pn-YBCO-BZO and spontaneously segregated YBCO nanocomposites (ss-nanocomposites). For pn-nanocomposite-BZO, it is shown a fairly constant ${J}_{c}^{self}$ (5 K) ~40 MA/cm^2^ up to 20 mol%, being the highest nanoparticle loading reported in epitaxial pn-YBCO nanocomposite with high critical current. This molar concentration approaches the optimal volume of secondary phases in PLD or MOCVD nanocomposite films having a nanorod structure^52–54^. Then, at 25 mol% loads the ${J}_{c}^{self}$ starts to decrease being in agreement with the texture degradation identified in the out-of-plane texture analysis above. By contrast, for ss- nanocomposites, a maximum ${J}_{c}^{self}$ (5 K) of 50 MA/cm^2^ was identified at 10 mol% and then rapidly decreased down to 10 MA/cm^2^ by increasing the nanoparticle load^24^. A critical temperature, T_c_, of ~90 K was obtained for the whole range of BZO concentrations, strengthening the assumption that the nanoparticles do not react with the YBCO. The effect of preformed BZO nanoparticles on the YBCO nanostructure (i.e. nanostrain ε, structural defect scenario) and the pinning performance will be evaluated in the next section.

#### Influence of nanoparticle composition and size on nanocomposite microstructure and pinning properties

On the basis of the above investigations, we focused our work on 20 mol% nanoparticles using a 25–50 nm YBCO seed layer and comparing first, two different compositions BZO and BHO, and then two different nanoparticle sizes: 10 and 5 nm of initial mean diameter.

**Figure 5:**
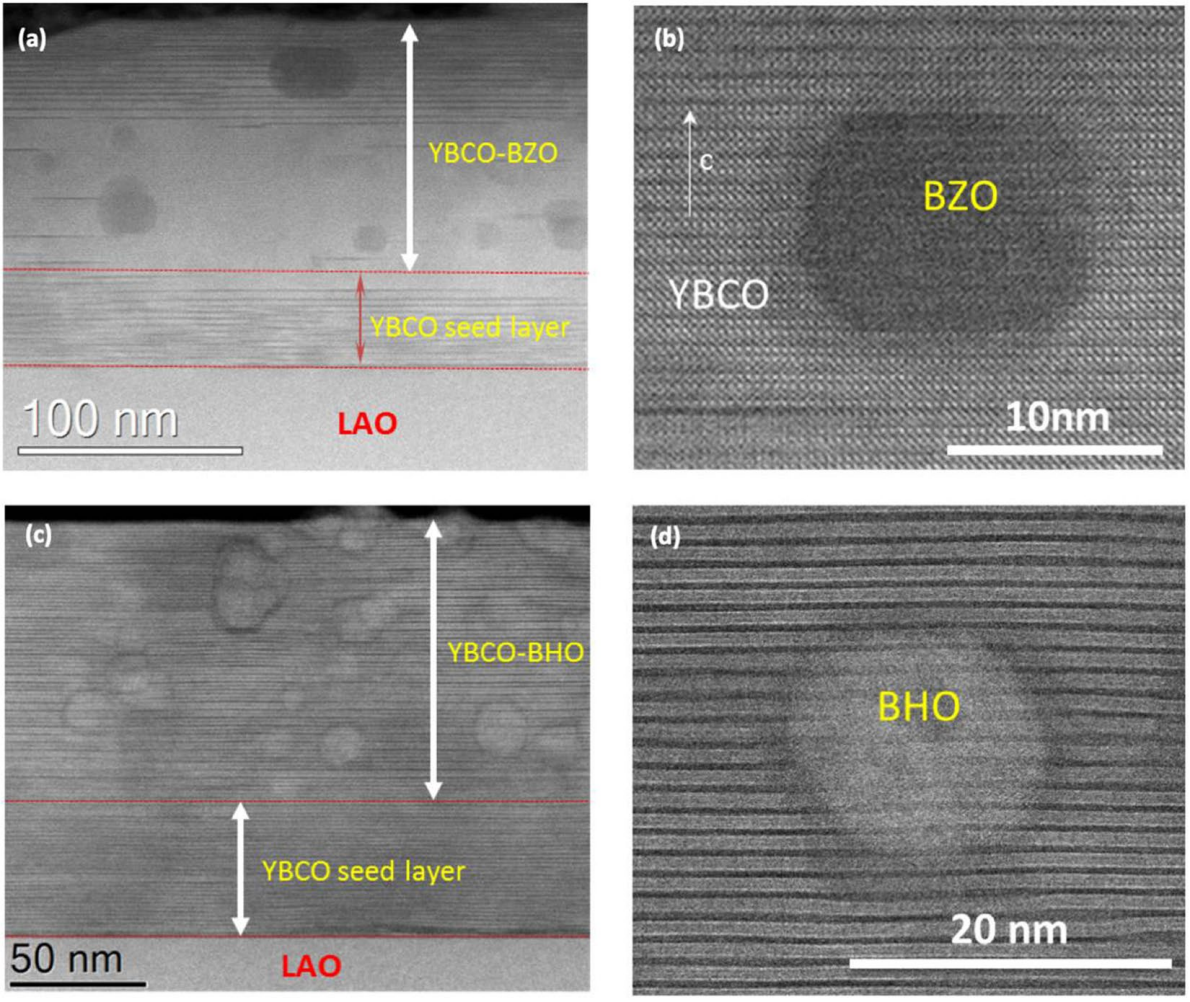
High magnification Z-contrast images of YBCO-20 mol% BMO (M = Zr and Hf, 10 nm) pn-nanocomposite films deposited on 50 nm YBCO seed layer. (**a**,**b**) YBCO-BZO composition and (**c**,**d**) YBCO-BHO.

Starting by nanoparticle composition, epitaxial YBCO pn-nanocomposites with 20 mol% BZO and BHO nanoparticles of 10 nm were prepared following the CTA (Figure S5). Texture analyses of the YBCO- pn-nanocomposite films show good in-plane (Δϕ ~ 0.8°) and out-of-plane (Δω ~ 0.2–0.4°) texture. In order to explore the nanoparticle distribution within the YBCO matrix and Y248 intergrowth scenario, STEM Z-contrast images were acquired. Figure 5(a) and (c) show the cross-sectional images of YBCO-BZO and YBCO-BHO pn-nanocomposites, respectively. In both cases, 50 nm pristine YBCO seed layer with high density of long intergrowths (up to 150 nm) are clearly identified. For YBCO-BZO, nanoparticles are homogeneously distributed within the nanocomposite layer although the intergrowths are mainly located in the upper part of the nanocomposite film and throughout the seed layer. By contrast, in YBCO-BHO, nanoparticles and long intergrowths are identified throughout the nanocomposite thickness. Isolated BZO and BHO nanoparticles embedded in the YBCO matrix are shown in Fig. 5(b) and (d), respectively. BZO are square-like and BHO are spherical with nanoparticle size of 15–20 nm. Therefore, the nanoparticles preserve the initial shape from the colloidal solutions (Fig. 1(c) and (d)) and undergo a limited nanoparticle size increase, from 10 nm to 20 nm. Therefore, the use of unreactive BMO_3_ nanoparticles allows to better control the nanoparticle distribution and coarsening than previously reported ss-nanocomposites or even pn-nanocomposites with reactive ZrO_2_, MFeO_4_ (M = Co, Mn) and CeO_2_ nanoparticles^41,42,55,56^.

In this scenario, the nanostrain obtained for YBCO-20 mol% BZO and YBCO-20 mol% BHO with initial size of 10 nm is ε = 0.18% and ε = 0.20%, respectively, in well agreement with the Y248 intergrowth scenario identified from STEM. Also, there is a significant increase from pristine YBCO (ε = 0.11%) and these values are similar to those obtained in optimal spontaneously segregated YBCO nanocomposites^24^.

**Figure 6:**
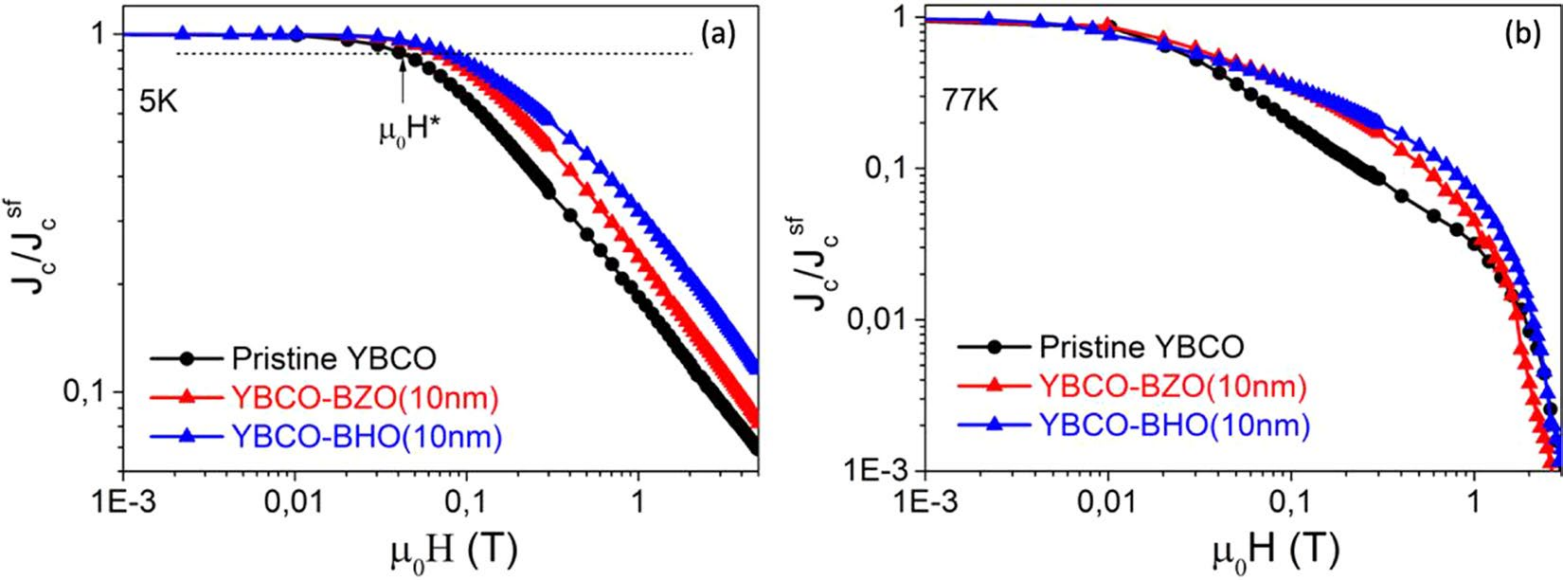
Magnetic field dependence of the critical current density at (**a**) 5 K and (**b**) 77 K normalized to its self-field value for pn-nanocomposite films with 20 mol% BZO (10 nm) (red triangle) and 20 mol% BHO (10 nm) (blue triangle) compared with a pristine YBCO (black circle). The horizontal dashed line in Fig. 5(a) marks the criterion to determine the *μ*_0_H* value $({{\rm{J}}}_{{\rm{c}}}({\mu }_{0}{\rm{H}}\ast )=0,9{{\rm{J}}}_{{\rm{c}}}^{{\rm{s}}{\rm{e}}{\rm{l}}{\rm{f}}})$.

High critical currents ${J}_{c}^{self}$ (77 K) of 4–4.5 MA/cm^2^ and T_c_ = 90 K are routinely obtained for these preformed 20 mol% BHO and BZO YBCO-nanocomposites. In order to evaluate the influence of nanoparticle composition on the pinning landscape, the magnetic field dependence of normalized critical current density ${{\rm{J}}}_{{\rm{c}}}/{{\rm{J}}}_{{\rm{c}}}^{{\rm{self}}}$ (H//c) has been investigated at two typical temperatures (5 and 77 K) and compared with pristine YBCO film, see Fig. 6. The crossover magnetic field, *μ*_0_H* (determined at 90% of ${{\rm{J}}}_{{\rm{c}}}^{{\rm{s}}{\rm{e}}{\rm{l}}{\rm{f}}}$), identifies the transition between single vortex pinning (regime in which vortices are pinned individually) to collective vortex pinning (vortex-vortex interactions start to be relevant and J_c_ decreases with the magnetic field)^23,34,57^. Here *μ*_0_H* at 5 K is shifted to higher fields for the nanocomposite samples (${\mu }_{0}{H}_{YBCO-BHO\,10\,nm}^{\ast }=71\,mT;\,{\mu }_{0}{H}_{YBCO-BZO\,10\,nm}^{\ast }=62\,mT\,$), compared to pristine films $({\mu }_{0}{H}_{YBCO}^{\ast }=38\,mT).\,$Thus, the nanocomposites show an increased pinning efficiency by the presence of higher amount of individual nanoscale defects which may well be the nanostrain generated due to the formation of the nanocomposite^24,40,41^. When comparing the two nanocomposite compositions, YBCO-BHO shows slightly higher *μ*_0_H* which could be ascribed to the higher values of nanostrain reported above. Overall, both nancomposites compositions are effective to improve the pinning landscape.

**Figure 7:**
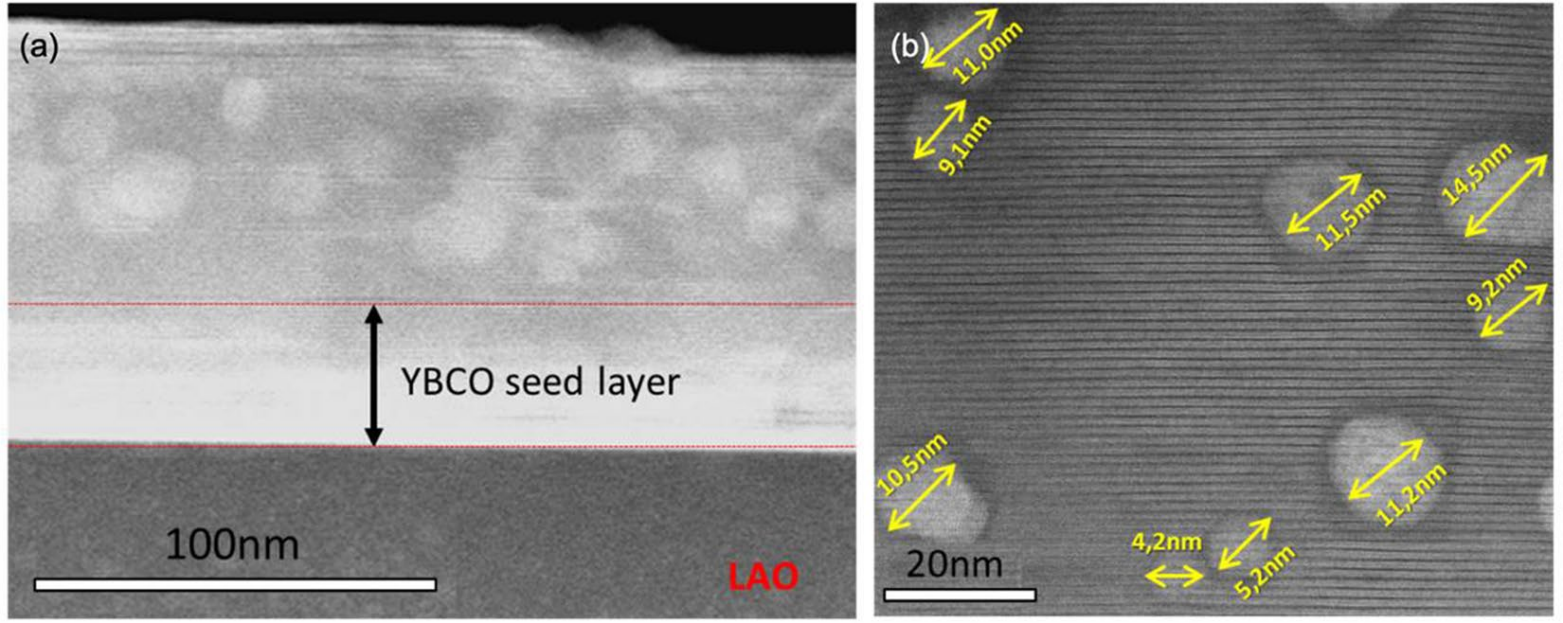
High resolution Z-contrast images of the YBCO-20 mol% BHO (5 nm) pn-nanocomposite. (**a**) Low resolution image of YBCO nanocomposite with embedded BHO nanoparticles; (**b**) high resolution image of mono dispersed BHO nanoparticles with limited coarsening and surrounded by high density of long stacking faults.

**Figure 8:**
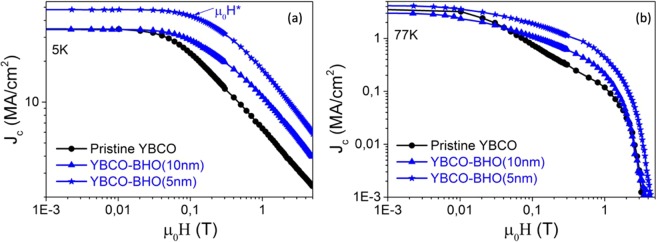
Critical current density versus magnetic field at (**a**) 5 K and (**b**) 77 K in YBCO-20 mol% BHO pn-nanocomposite films with initial nanoparticle size of 10 nm (blue triangle) and 5 nm (blue star) compared with a pristine YBCO film (black circle).

In order to study the influence of the initial nanoparticle size (5 nm vs 10 nm) on the final microstructure and pinning performance of the YBCO pn-nanocomposite, YBCO-20% mol BHO composition is selected and processed following CTA. High resolution Z-contrast images of pn-YBCO-20 mol% BHO with 5 nm initial size are shown in Fig. 7. Homogeneously distributed BHO nanoparticles are identified throughout the nanocomposite thickness along with high density of Y248 intergrowths with at least 80 nm of lateral extension. Closer look at the embedded nanoparticles shows that the mean particle diameter increased up to an average size of 10 nm, see Fig. 7b. This nanoparticle size has also been confirmed by XRD using the Debye-Scherrer equation. Hence, starting from smaller nanoparticles, the final diameter is also increased by a factor of two although they remain as discrete nanoparticles. Texture analysis reveals a biaxially textured superconducting matrix with Δϕ (103) YBCO = 0.9^◦^ and Δω (005) YBCO = 0.5°. The nanostrain of these YBCO-20 mol% BHO (5 nm) pn-nanocomposites is ε = 0.21%, similar to the analogous YBCO-BHO pn-nancomposites (10 nm) films. Figure 8 compares the J_c_(H//c) dependence for YBCO pn-nanocomposite with 10 nm and 5 nm initial mean diameter at (a) 5 K and (b) 77 K. Noteworthy, the YBCO-BHO nanocomposites with smaller diameter size (5 nm) show an overall J_c_ enhancement for the whole range of magnetic field here studied. Remarkable critical current densities of ${{\rm{J}}}_{{\rm{c}}}^{{\rm{s}}{\rm{e}}{\rm{l}}{\rm{f}}}$(77 K) = 4.5 MA/cm^2^ and ${{\rm{J}}}_{{\rm{c}}}^{{\rm{s}}{\rm{e}}{\rm{l}}{\rm{f}}}$(5 K) = 48 MA/cm^2^ are obtained. Furthermore, a softer magnetic field decay appears for the 5 nm YBCO-BHO nanocomposites with *μ*_0_ ${H}_{YBCO-BHO\,5\,nm}^{\ast }=100\,mT,\,$indicating that smaller nanoparticles help increasing the pinning efficiency at applied magnetic fields.

**Figure 9:**
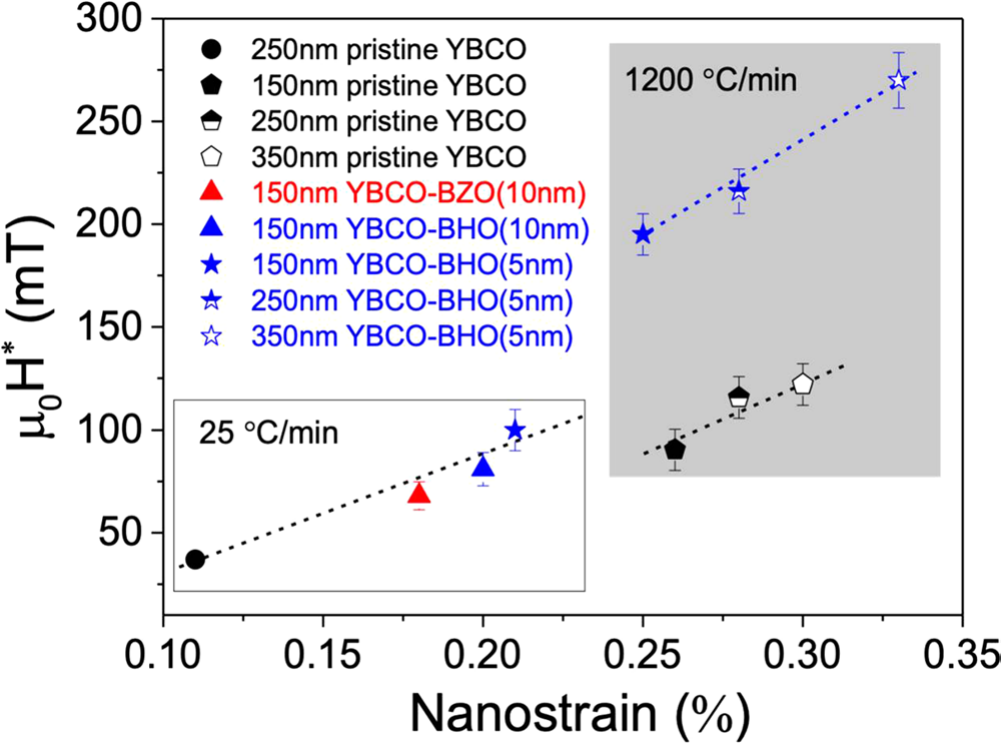
Dependence of the *μ*_0_H* at 5 K on the nanostrain for pristine YBCO and YBCO-BMO pn-nanocomposites comparing composition (BHO vs BZO) and size (5 nm vs 10 nm) of the nanoparticles in CTA (25 °C/min) and for the FH (1200 °C/min) at different thickness (150 nm, 250 nm and 350 nm).

When looking at the *μ*_0_H* dependence with nanostrain for the CTA (25 °C/min), Fig. 9 left side, it is easily identified that the incorporation of nanoparticles in the YBCO leads to a large increase in the nanostrain and consequently larger *μ*_0_H*. Therefore, the chemical stability and the small nanoparticle size are both key parameters to drive the formation of Y248 intergrowths in the YBCO matrix and thus modulate the nanostrain.

#### Nanoparticle coarsening and intergrowth control: flash heating conversion process

In order to further tune the formation of Y248 intergrowths and better control the nanoparticle size of the preformed nanoparticles during the YBCO growth, an improved thermal treatment has been designed with fast heating ramps (1200 °C/min), named flash heating (FH) (see Figure S1(a)). This thermal profile enables epitaxial growth of pristine YBCO thin films in a wider range (750 °C–810 °C) with reduced porosity. The FH process in pristine YBCO leads to T_c_ ≈ 89 K and ${J}_{c}^{self}$ (77 K) = 2–3 MA cm^−2^. This is attributed to the presence of secondary phases such as Y_2_Cu_2_O_5_ (Y225), which are well known to form during the conversion mechanism of YBCO, see Figure S6^47^.

**Figure 10:**
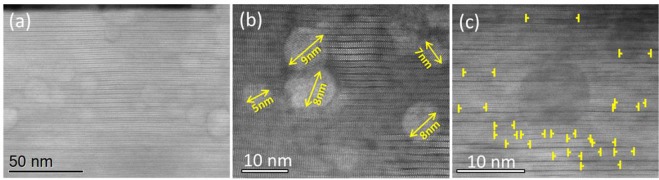
STEM images of YBCO-20 mol% BHO (5 nm) pn-nanocomposite processed following with flash heating at 750 °C. (**a**) Low-resolution image showing a general view of the YBCO pn-nanocomposite layer and the seed layer; (**b**) high-resolution image of BHO nanoparticles with minimal coarsening within the YBCO matrix. (**c**) High-resolution image of an isolated BHO surrounded by short intergrowths (indicated by yellow arrows).

The effect of the FH thermal process on the YBCO pn-nanocomposite structure and pinning properties has been investigated for the YBCO-BHO composition. Biaxially textured YBCO -pn- nanocomposites with Δϕ (103) YBCO = 1.1^◦^ and Δω (005) YBCO = 0.5^◦^ are routinely obtained. Note that in this case the Δϕ is increased compared to the CTA samples which could be attributed to the presence of secondary phases such Y225 and different scenario of structural defects. Figure 10 shows Z-contrast images of YBCO-20% mol BHO (5 nm) film grown at 750 °C. From the general overview image, in Fig. 10(a), the film shows the typical microstructure identified in the previous samples: pn-nanocomposite film full of intergrowths where the nanoparticles are homogeneously distributed within the superconducting matrix. Deeper insight, Fig. 10(b), reveals that the nanoparticle mean diameter after the YBCO growth (7 nm) barely differs from the diameter of the BHO nanoparticles in the colloidal solution (Figure S7), also confirmed by the Debye Scherrer equation. Along with this important finding, it is observed that the Y248 intergrowths surrounding the nanoparticles show a much shorter lateral extension, Fig. 10(c), than the pn-nanocomposites processed under CTA^41,42^.

**Figure 11:**
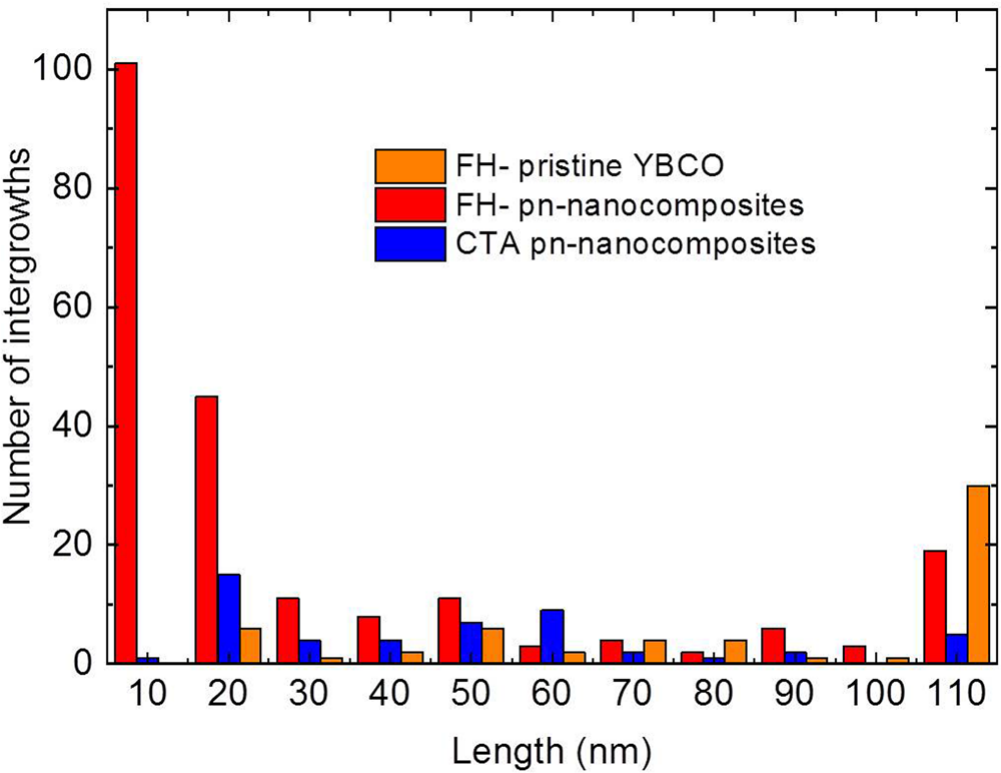
Histogram showing the length and number of intergrowths for FH and CTA process in pristine and pn-nanocomposite films.

From the STEM images of pn-nanocomposites processed under CTA and FH, and pristine FH (Figure S8) it has been calculated the length and number of intergrowths generated in the YBCO matrix. As shown in Fig. 11, FH- samples lead to higher amount of intergrowths compared to CTA. Note that FH-nanocomposites display the highest amount of intergrowths being most of them short. Even though, the FH- pristine films have longer intergrowths than the nanocomposite CTA films (Fig. 11), thus contributing to a larger nanostrain, this does not transform in a corresponding increase on *μ*_0_H* because the volume of partial dislocations remains unchanged as explained below.

Strain analysis combined with different STEM imaging modes confirmed that the lattice deformation of the YBCO matrix is localized around the partial dislocations that surrounds the intergrowths and this local deformation may be extended up to a volume of several nanometers (see Figure S9)^32^. The dislocation volume can be calculated as^58^

$${{\rm{V}}}_{{\rm{i}}}={\rm{\pi }}({{\rm{R}}}_{{\rm{i}}}+{\rm{\delta }}/2)2{\rm{\delta }}-{\rm{\pi }}({{\rm{R}}}_{{\rm{i}}}-{\rm{\delta }}/2)2{\rm{\delta }}=2{{\rm{\pi }}{\rm{R}}}_{{\rm{i}}}{\rm{\delta }}2$$

where *V*_*i*_ is the volume of each dislocation, *R*_*i*_ the radius of the stacking fault and *δ* is the size of the strained region caused by the dislocation (≈0.8 nm). Then, to compute the dislocation volume percentage, *ρ*_*i*_, we take into consideration the volume of the *n* dislocations found in a well-defined volume image (Δx, Δy, Δz)

$${\rho }_{dis}=\frac{{\sum }_{i}^{n}{V}_{i}}{{{\rm{\Delta }}}_{x}{{\rm{\Delta }}}_{y}{{\rm{\Delta }}}_{z}}$$

From this equation it has been calculated the ρ_dis_ for FH BHO 5 nm (2.3%) and CTA BHO 5 nm (0.7%). Therefore, FH process in nanocomposites offers several advantages over the CTA: faster processing, preserves small nanoparticle size and generate higher density of partial dislocations where the intergrowths are much shorter being thus very promising to improve the vortex pinning of the nanocomposites.

**Figure 12:**
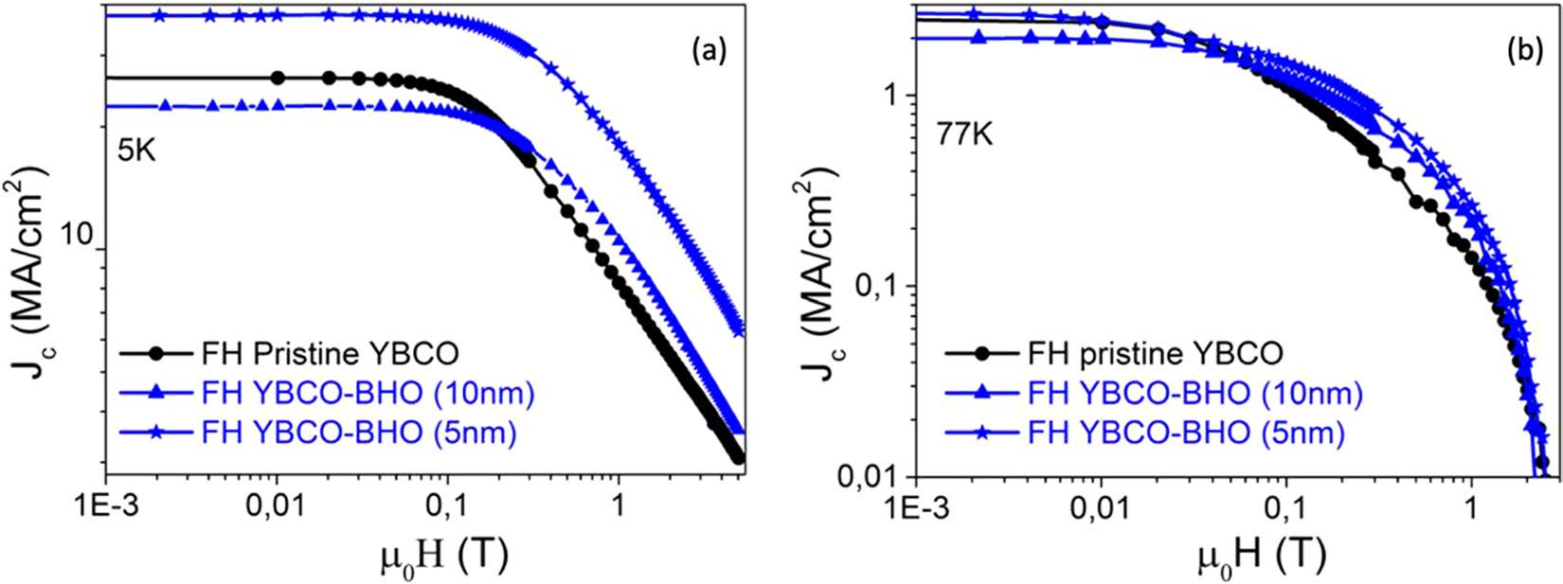
Magnetic field dependence of the critical current density J_c_(H) measured at (**a**) 5 K and (**b**) 77 K of YBCO-20 mol% BHO pn-nanocompostite films with initial nanoparticles size of 10 nm (blue triangle) and 5 nm (blue star) compared with pristine YBCO thin films (black circle). All the samples are grown using FH.

Figure 12 presents the J_c_(H) dependence of pristine YBCO, YBCO-20 mol% BHO (10 nm) and YBCO-20 mol% BHO (5 nm) pn-nanocomposite films grown by FH and measured at (a) 5 K and (b) 77 K. Once again the nanocomposite samples present a much smoother J_c_(H) dependence compared to the pristine YBCO being the 5 nm nanoparticle nanocomposite much better than the 10 nm. Indeed, the *μ*_*o*_ ${H}_{FH-YBCO-BHO\,5\,nm}^{\ast }$ is 195 mT, similar to the best performances reported up to date in solution deposited films^23^. At this stage, when looking at the *μ*_*o*_H* dependence with nanostrain, Fig. 9 - right side (1200 °C/min), it is observed that the nanostrain of the FH films are all shifted to higher nanostrain consistent with the fact that FH samples show higher ρ_dis_ than samples processed under CTA conditions. The nanostrain of 150 nm FH-films, independently of being nanocomposite or pristine film is ε ≈ 0.25%, nonetheless, the *μ*_*o*_H* is significantly higher for the nanocomposite. Therefore, besides higher ρ_dis_ with shorter intergrowths, there might be a new added contribution to the *μ*_*o*_H* of nanocomposites.

**Figure 13:**
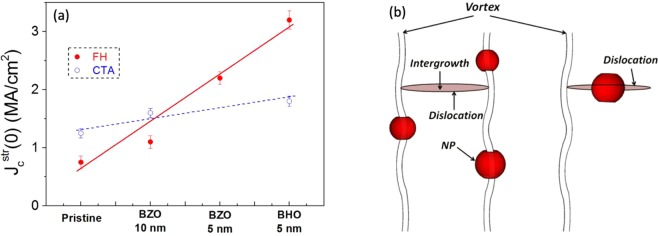
Strong pinning contribution in YBCO pn-nanocomposites. (**a**) Strong pinning contribution ${J}_{c}^{str}$ at 0 K obtained from fitting J_c_(T) dependence at 7 T for pristine and pn-nanocomposites prepared from CTA and FH thermal process. (**b**) Sketch of vortex pinning of both small nanoparticles (NP) and partial dislocations.

From the temperature dependence of J_c_ it is possible to classify the strength of the active artificial pinning centers according to their thermal activation process^21,34^. We can differentiate two contributions: strong pinning, ${J}_{c}^{str}$, which shows a smooth decay with temperature (i.e. nanostrain, planar defects (intergrowths), twin boundaries, nanoparticles or nanorods with diameter size in the range of the coherence length, ξ) $({J}_{c}^{wk}(T)={J}_{c}^{wk}(0)exp(\,-\,T/{T}_{0}))\,$and weak pinning, ${J}_{c}^{wk}$, which is characterized by a fast J_c_ decay with temperature (i.e. point defects such as oxygen vacancies and atomic substitutions) (${J}_{c}^{str}(T)={J}_{c}^{str}(0)exp[\,-\,3{(T/{T}^{\ast })}^{2}]$), see Figure S10. Figure 13(a) shows the contribution of ${J}_{c}^{str}$ at 0 K and μ_0_H = 7 T, obtained by fitting the weak and strong *J*_*c*_(*T*) dependences. We compare pristine YBCO, pn-nanocomposite with BZO (10 nm), BZO (5 nm) and BHO (5 nm). While the ${J}_{c}^{wk}$ (0) contribution is not directly correlated to the nanoparticle concentration^34^, The presence of nanoparticles enhances the strong pinning contribution in both processes, as expected due to the large nanostrain obtained in pn-nanocomposites, which act as strong pinning defect^24^. It is worth noting that the samples flash heated with small nanoparticle size (5 nm) presents the highest ${J}_{c}^{str}(0)\,$values. With the FH process the size of the nanoparticle in pn-nanocomposite films is preserved and thus a final diameter of 5–7 nm is obtained. In this case, it is very likely that the nanoparticles themselves would be synergistically associated to the partial dislocations to enhance vortex pinning, because their mean diameter is close to the coherence length (ξ) (Fig. 13(b)). Importantly, up to date, this scenario had been only clearly reported for vacuum deposited films where such small diameter of nanoparticles or nanorods could be achieved^4,23,29,52,59–61^. Besides, the density of 5 nm nanoparticles, n_np_ in FH films (n_np_ ≈ 40 × 10^22^ m^−3^) increased by a factor two compared to CTA nanocomposites (n_np_ ≈ 14 × 10^22^ m^−3^) which corresponds to a 7.7% in volume of nanoparticles. This density is similar to the recently reported high performance ss- nanocomposites with high load nanoparticles and tunable size^23^.

#### Multideposition to modulate YBa_2_Cu_3_O_7−X_ nanocomposite thickness

**Figure 14:**
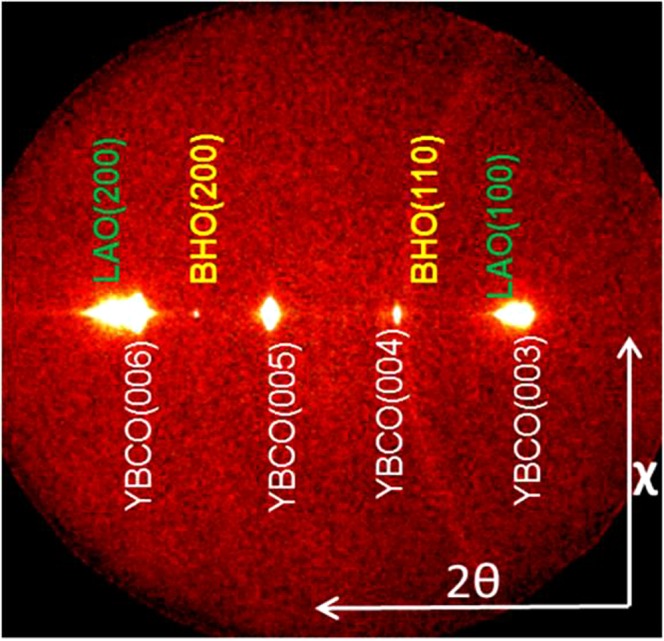
2D θ-2θ XRD analysis of 350 nm YBCO −20 mol% BHO (5 nm) pn-nanocomposite layer deposited on a 50 nm YBCO buffered LAO substrate grown from FH.

**Figure 15:**
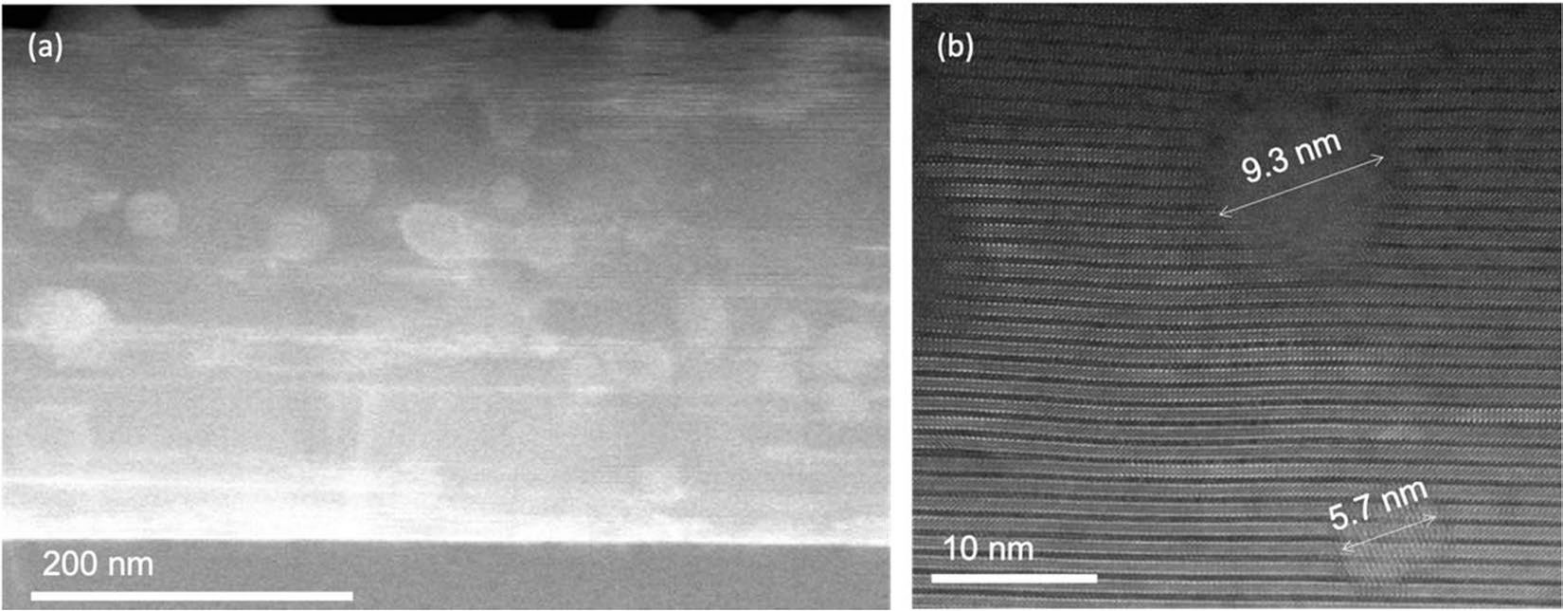
Z-contrast images of the 350 nm YBCO-20 mol% BHO (5 nm) pn-nanocomposite film grown from flash heating process at 750 °C. (**a**) Low resolution image of the overview of the pn-nanocomposite film. (**b**) high resolution image of the BHO nanoparticle embedded inside in the highly bended YBCO matrix.

Based on the success of the results illustrated in the previous sections, the effort has been then focused on FH multideposition process to modulate pn-nanocomposite thickness using YBCO-20% mol BHO (5 nm) (details in section 2.2). A typical 2D XRD θ-2θ pattern of 350 nm nanocomposite film is displayed in Fig. 14. The YBCO film is epitaxial and the BHO secondary phase is identified at 2θ = 30.2° as a strong ring and at 2θ = 43.4° as a faint spot corresponding to BHO (110) and BHO (200) Bragg reflections, respectively. In 350 nm film, we are above the minimum detectable amount of BHO to differentiate the ring. The percentage of randomly oriented nanoparticles, key feature to ensure high nanostrain in CSD nanocomposites^24^, is estimated to be 94% in YBCO-BHO nanocomposite (see Figure S11), similar to the results obtained in optimal ss-YBCO nanocomposites^22,36^. The multideposited films (150, 250 and 350 nm, Figure S12) still preserve biaxial texture with FWHM ϕ-scan (103) values of 0.7°, 1.01° and 1.45° and FWHM of ω-scan = 0.52°, 0.63° and 0.73°, respectively. From the high resolution Z-contrast images of the 350 nm films, Fig. 15, it can be extracted that the nanoparticles preserve the original diameter size and are homogeneously distributed within the YBCO matrix. No layer boundaries neither the appearance of CuO segregation between the layers have been identified in this multideposited samples^62^. Therefore, by increasing the film thickness a factor of 2 the YBCO structural defect scenario and embedded nanoparticle characteristics are essentially preserved. The nanostrain has also been calculated for the multilayered systems identifying a clear increase from 0.25% to 0.33%, being the latter the highest obtained in preformed nanocomposites^41,42^. The *μ*_0_H* at 5 K in these nanocomposites is continuously increased with film thickness, from 195 mT (150 nm) to 216 mT (250 nm) and ultimately to 270 mT (350 nm), indicating a continuous increase of vortex pinning efficiency at higher magnetic fields. This would suggest that the single vortex pinning regime (determined by *μ*_0_H*) is extended towards higher fields probably because by increasing the film thickness there is an increase of the density of short intergrowths and so of the partial dislocation total density while the final nanoparticle size is preserved. Further microstructural and physical properties analysis would be necessary to sort out the relative weight of each contribution.

**Figure 16:**
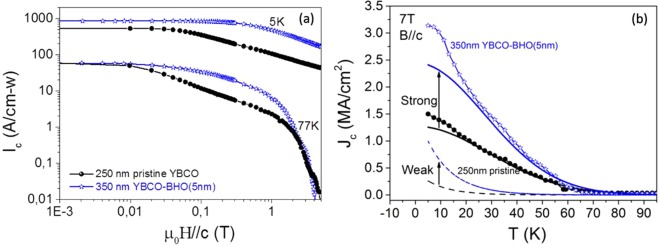
(**a**) Magnetic field dependence of the total critical current, I_c−w_(H), for 250 nm pristine YBCO (black line) and 350 nm YBCO-20 mol% BHO (5 nm) pn-nanocomposite (blue line) measured at 5 K and 77 K (SQUID), (**b**) temperature dependence of the critical current density J_c_ (T) measured at 7 T for the pristine YBCO and 350 nm YBCO-20 mol% BHO (5 nm) pn-nanocomposite. The curves have been fitted with two contributions (strong and weak pinning). The 250 nm pristine YBCO film is processed by conventional thermal treatment and the 350 nm pn-nanocomposite following the flash heating process.

Figure 16(a) shows the magnetic field dependence of the total critical current (I_c-w_) for 250 nm pristine YBCO and 350 nm preformed BHO nanocomposite, measured at 5 and 77 K. The nanocomposite shows a smoothed magnetic field dependence. Actually, the I_c-w_ increases linearly by increasing the film thicknesses in pn-nanocomposite films (see Figure S13). Figure 16(b) shows the J_c_(T) dependence measured under a constant field of 7 T for 250 nm pristine YBCO film and 350 YBCO-BHO preformed nanocomposite films. It is observed that the nanocomposite curve is well above the pristine YBCO and the major contribution of this enhancement comes from the strong pinning contribution (blue solid line), although the weak contribution is also slightly increased in the nanocomposite (blue dashed line). Thus, the key features achieved in a single deposition pn-nanocomposite are maintained in moderate thicknesses with an overall enhancement of the superconducting performances.

## Conclusions

We have demonstrated the versatility of solution deposited YBCO nanocomposites with preformed perovskite nanoparticles to tune the YBCO microstructure and further tailor the pinning performance in applied magnetic field. A progressive optimization has been carried out by tight control of the following processing parameters for improved superconducting and pinning properties: (1) the use of a pristine YBCO seed layer prior to pn-nanocomposite solution deposition to achieve epitaxial nanocomposites, (2) concentration, size and composition of preformed BMO nanoparticle to minimize coalescence and generate high density of intergrowth, (3) growth heating ramps to modulate the kinetics of the process (flash heating versus conventional heating) and (4) multideposition process.

The introduction of a 25–50 nm pristine YBCO seed layer prior nanocomposite solution deposition is beneficial to ensure epitaxial growth of the pn- nanocomposite for nanoparticle composition starting from 12 mol%. Strongly improved YBCO properties have been achieved by incorporating up to 20 mol% of 5 nm BHO nanoparticles due to the generation of high density of intergrowth and consequently increased nanostrain. Flash heating is a fast process which has been proved efficient to grow epitaxial nanocomposites in a wider growth temperature window while preserving discrete and small nanoparticles and simultaneously generate high density of intergrowth. These two key parameters play a synergistic effect to increase the artificial pinning centers and enhance the vortex (strong) pinning landscape. Multideposition process is validated to further increase the nanocomposite thickness and further enhance the vortex pinning efficiency and total critical current carrying capability.

Finally, the processing optimization here presented is already a noteworthy improvement compared to previous spontaneously segregated and preformed nanocomposites that can be extended to a wider variety of REBCO (RE = rare earth) superconductors and it is foreseen to be compatible with coated conductors.

## Supplementary information

SUPPORTING INFORMATION
