# Supplementary material for: Control of nanostructure and pinning properties in solution deposited YBa2Cu3O7−x nanocomposites with preformed perovskite nanoparticles
Source: Sci Rep. 2019 Apr 9;9:5828. doi: 10.1038/s41598-019-42291-x (PMC6456625; doi:10.1038/s41598-019-42291-x)
Supplement: Supplementary file 1 — SUPPORTING INFORMATION [file 41598_2019_42291_MOESM1_ESM.docx]

## **Supplementary Information for**

## **Control of nanostructure and pinning properties in solution deposited YBa_2_Cu_3_O_7-x_ nanocomposites with preformed perovskite nanoparticles**

Ziliang Li^1^, Mariona Coll^1^*, Bernat Mundet^1^, Natalia Chamorro^2^, Ferran Vallès^1^, Anna Palau^1^, Jaume Gazquez^1^, Susagna Ricart^1^, Teresa Puig^1^, Xavier Obradors^1^*

^1^Institut de Ciència de Materials de Barcelona, CSIC, Campus de la UAB, 08193 Bellaterra, Spain

^2^Departament de Química, Facultat de Ciències, Universitat Autònoma de Barcelona, 08193-Cerdanyola del Vallès, Catalonia, Spain


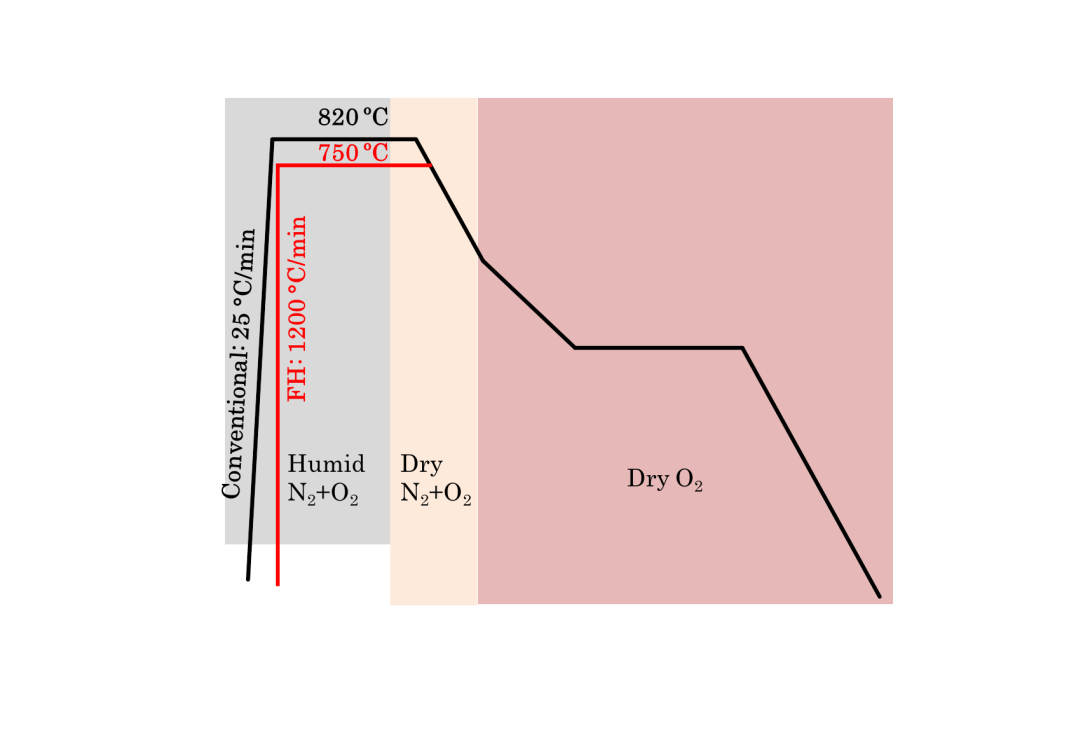


(b)

(a)


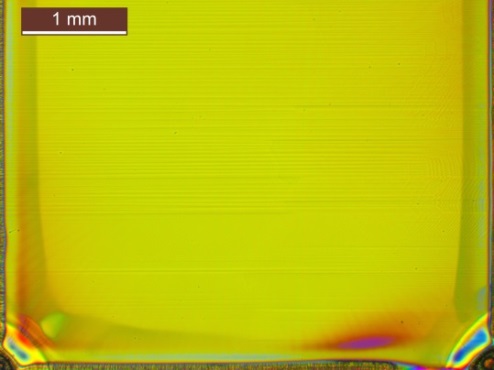

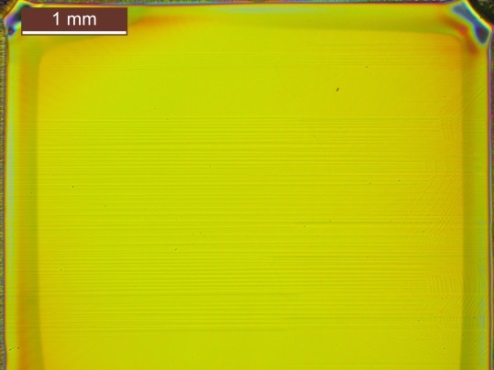


**Figure S1 (**a) Heating profiles used in this work to convert the YBCO pn-nanocomposite thin films: Conventional process (CTA): 25 ºC/min, T= 820 ºC ; flash heating process (FH): 1200 ºC/min, T= 750 ºC; (b) Typical optical microscope image of as-pyrolyzed YBCO-BMO nanocomposite films showing homogeneous and crack free surface.


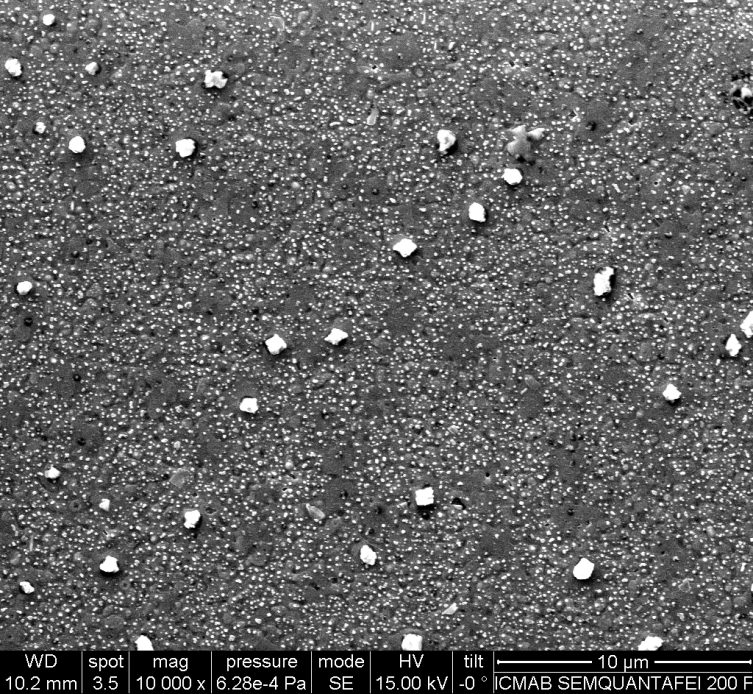

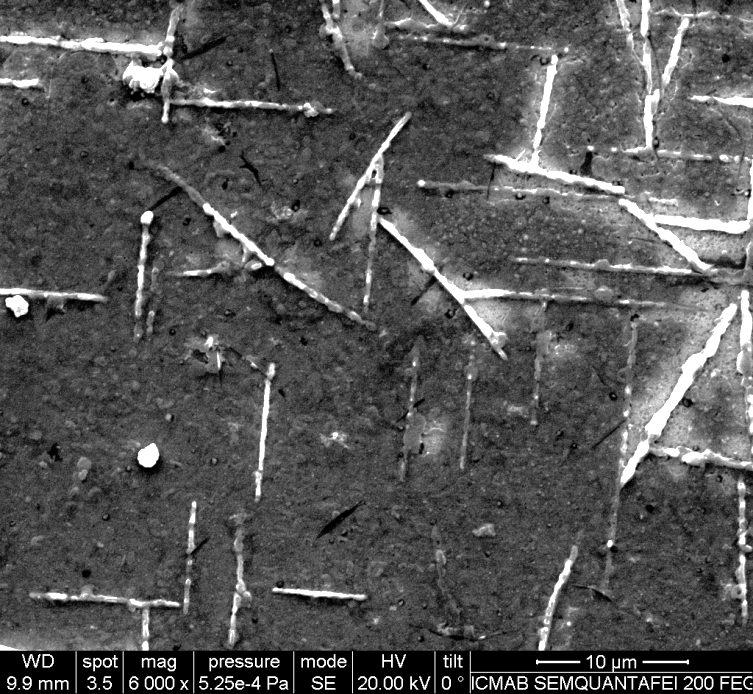


(a)

(b)

**Figure S2** SEM images of (a) YBCO-6 mol% BZO (10 nm) and (b) YBCO-12 mol% BZO (10 nm) pn-nanocomposite deposited on bare LAO single crystal and processed following the CTA.

For YBCO-6% mol BZO c-axis YBCO with few surface precipiates (Ba-Cu-O rich) are observed. For YBCO-12 mol% BZO, random YBCO grains start to appear in the c-axis oriented YBCO film and YBCO seed layer is required to ensure full c-axis growth.

**
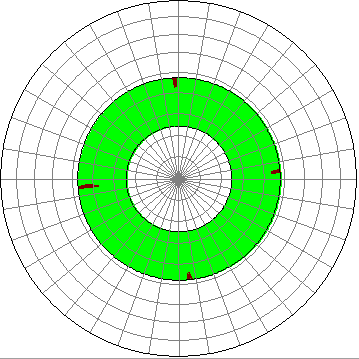

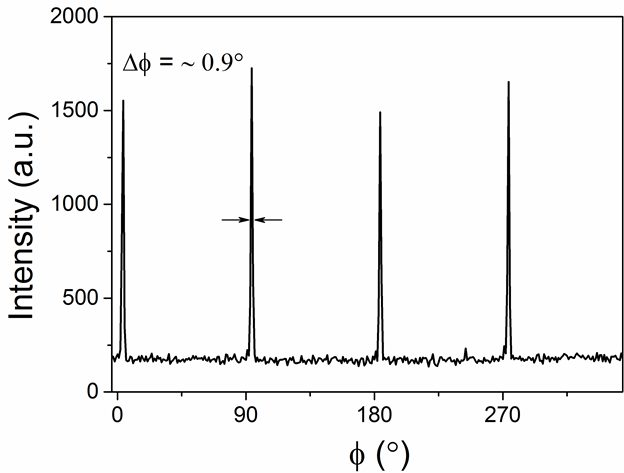
**

(a)

(b)

**Figure S3** (a) X-ray pole figure of (103) YBCO and (b) phi-scan of the (103) YBCO reflection preformed in YBCO-20 mol% BZO (10 nm) pn-nanocomposite film grown from CTA indicating good in-plane texture.


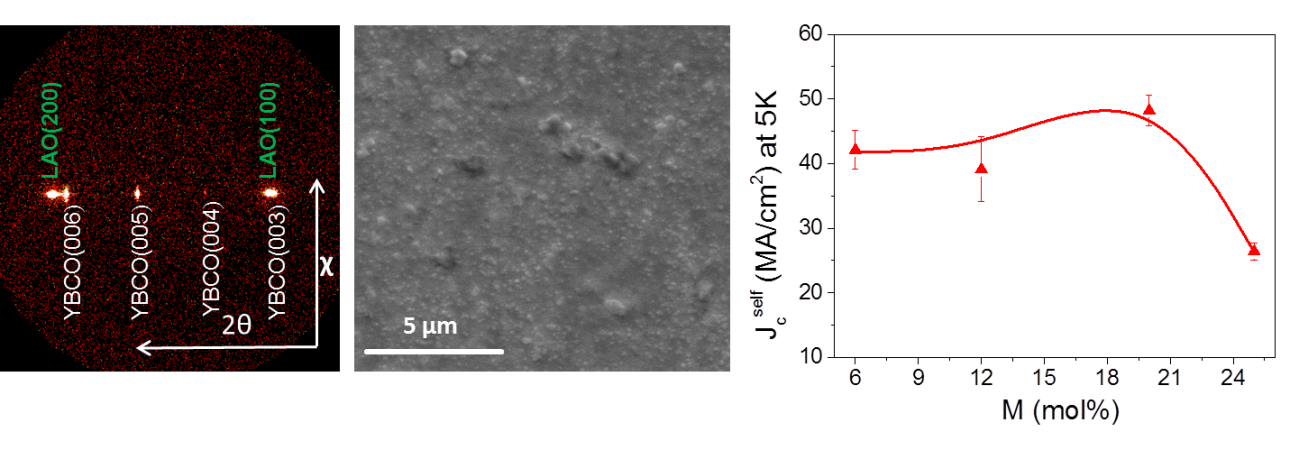


(a)

(b)

**Figure S4** (a) 2D θ-2θ XRD diffraction patterns of YBCO-25mol% BZO (10nm) pn-nanocomposite on 50 nm pristine YBCO//LAO single crystal and grown from CTA, (b) corresponding SEM image of the sample in (a). c-axis YBCO films with absence of randomly oriented grains can be obtained with concentrations of BZO as high as 25 mol%.


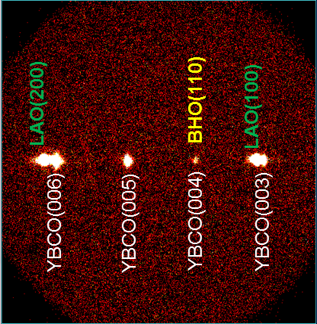


(a)

(b)

**Figure S5** (a) Representative 2D θ-2θ XRD pattern of a YBCO-20mol% BHO nanocomposite grown on 25-50 nm pristine YBCO buffered LAO substrate from CTA. (b) Integrated spectrum of (a). The (110) Bragg reflection of BHO confirms the presence of nanoparticles within the epitaxial (00l) YBCO films.

**Figure S6** XRD θ-2θ scan of YBCO + 20 mol% BHO (5 nm) grown from the flash heating process. Y_2_Cu_2_O_5_ (Y225) secondary phase is identified due to the fact that the film undergoes a different kinetic process than with CTA. The formation of Y225 competes with the formation of YBCO and the fast processing seems to favor small amount of Y225 formation.


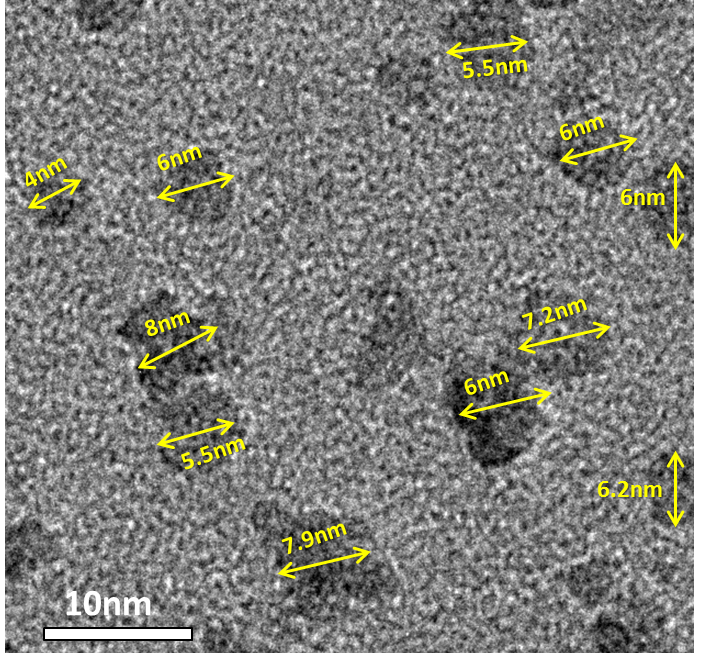


**Figure S7** Bright field TEM image of preformed BHO nanoparticle colloidal solution. Nanoparticle diameter has been marked by yellow arrows (5-7 nm of mean diameter size).


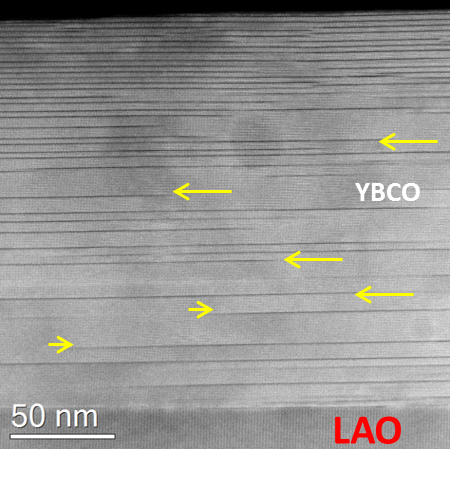


**Figure S8** STEM image of prisitine YBCO thin film grown from FH process at 750 ºC. Yellow arrows indicate the intergrowths formed during the YBCO. Flash heating favors the formation of intergrowths compared to CTA.


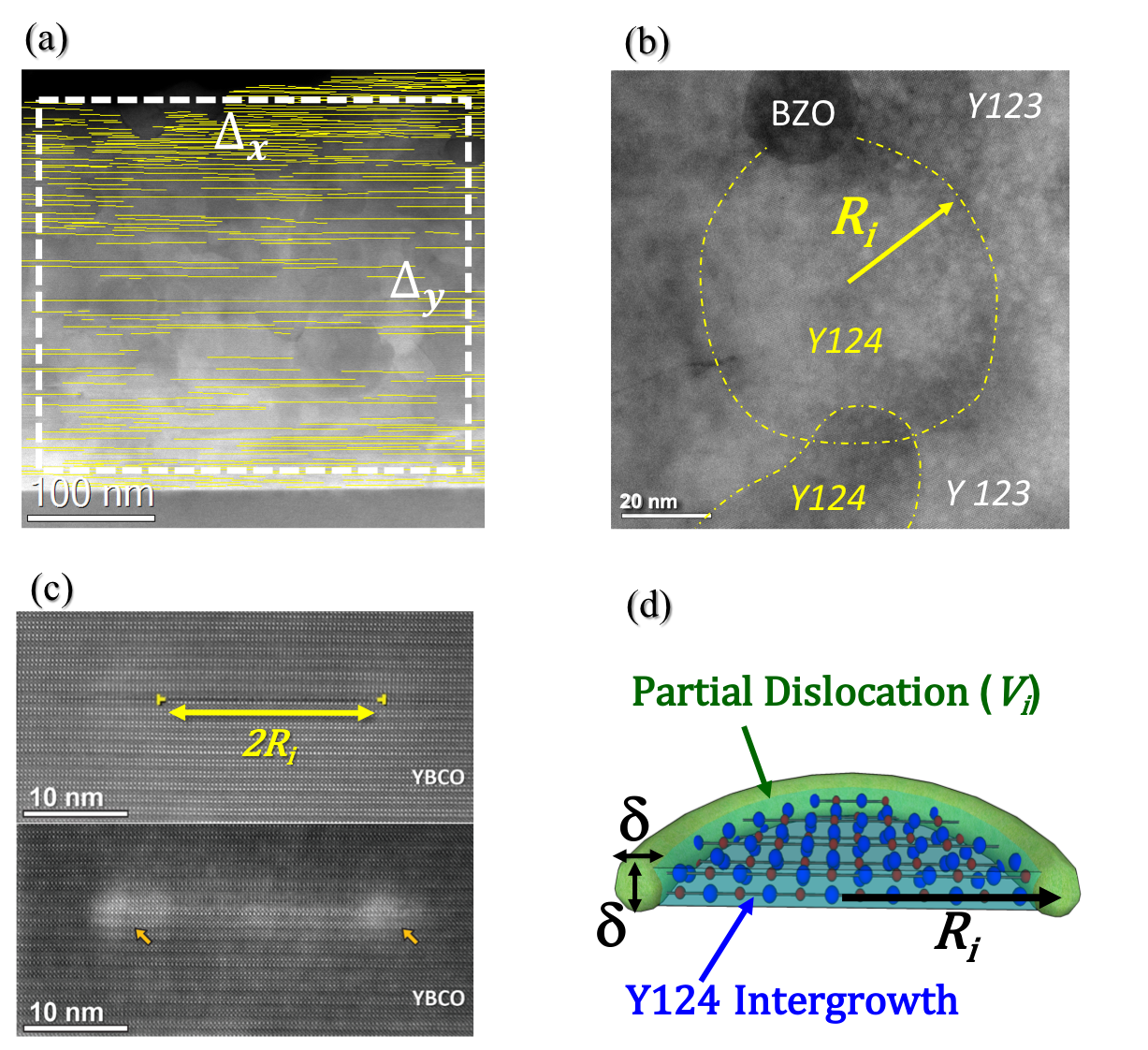


**Figure S9** : STEM observation of stacking faults in YBCO nanocomposite films and schematic descriptions of the defect. (a) Cross-sectional low magnification Z-contrast images of solution deposited YBCO nanocomposite where stacking faults are marked with yellow stripes and the observed YBCO area ∆x∆y is delimited. (b) Plan view low magnification Z-contrast image of a solution deposited YBCO nanocomposite. The boundaries of intergrowths are highlighted in yellow. (c) Z-contrast (above) and Low-Angle Annular Dark Field (below) images of an isolated 25 nm-long stacking fault. The yellow symbols in the images point to partial dislocations while the arrows to their surrounding strain fields, with size δ = 0.8nm . (d) Schematic view of the partial dislocation surrounding the stacking fault.

**Figure S10** J_c_ (T) for FH- YBCO pristine film of 150 nm thickness. Weak and strong contributions have been fitted with $J_{c}^{wk}(T)=J_{c}^{wk}\left( 0 \right)exp\left( \frac{-T}{T_{0}} \right)$and $J_{c}^{str}(T)=J_{c}^{str}\left( 0 \right)exp\left[ -3\left( \frac{T}{T^{*}} \right)^{2} \right]$, were the fitting parameters $J_{c}^{wk}\left( 0 \right)$ and $J_{c}^{str}\left( 0 \right)$ are the contributions to *J_c_* at 0K of weak and strong pinning defects, respectively, and *T_0_* and *T^*^* are their characteristic pinning energies.


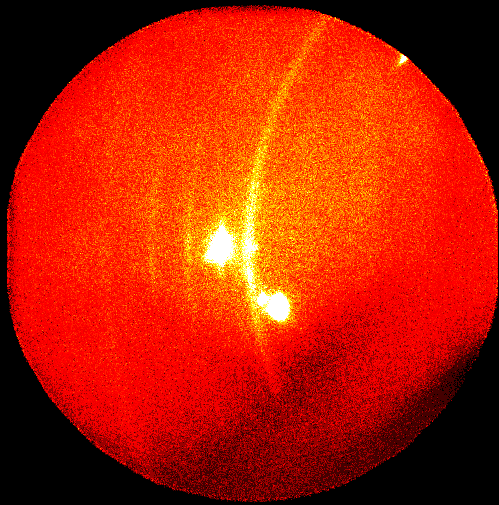


BHO (110)

YBCO (103)

YBCO (102)

**Figure S11**. 2D- XRD pattern centered at the (110) Bragg reflection of the BHO nanodots (Chi =45º) allowing the quantification of the fraction of the randomly orientated nanodots.


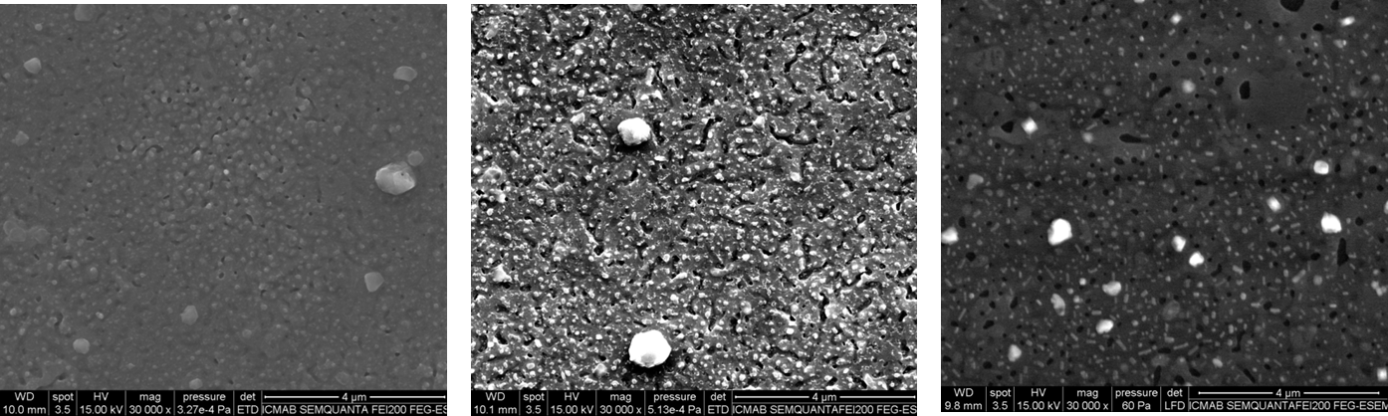


(c)

(b)

**Figure S12** SEM images of a) YBCO-20 mol% BHO (5nm) pn-nanocomposite single layer, b) YBCO-20 mol% BHO (5nm) pn-nanocomposite bilayer and YBCO-20 mol% BHO (5nm) PN-nanocomposite trilayer. All the nanocomposite films are deposited on 25-50nm pristine YBCO-buffered LAO substrate and grown from FH (750 ºC) process. The films present an homogeneous surface with some typical Ba-Cu-O surface precipitates and no traces of random YBCO grains.


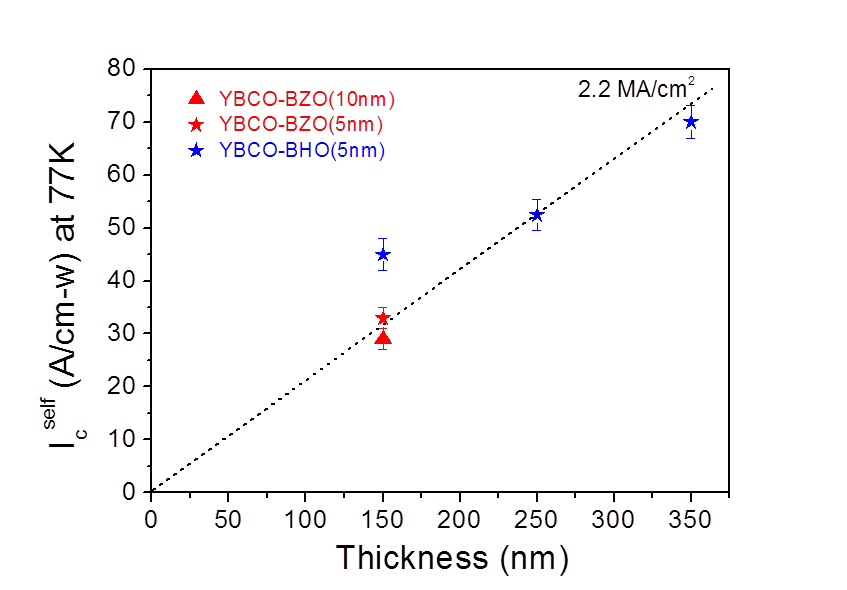


**Figure S13** Critical current per cm in width (I_c-w_) evolution with films thickness of YBCO-BMO (M=Zr and Hf) grown from the flash heating process measured in self-field at 77K. The I_c_ increases linearly by increasing the film thickness.
